# Concurrent micro- to macro-cardiac electrophysiology in myocyte cultures and human heart slices

**DOI:** 10.1038/s41598-018-25170-9

**Published:** 2018-05-02

**Authors:** Rasheda A. Chowdhury, Konstantinos N. Tzortzis, Emmanuel Dupont, Shaun Selvadurai, Filippo Perbellini, Chris D. Cantwell, Fu Siong Ng, Andre R. Simon, Cesare M. Terracciano, Nicholas S. Peters

**Affiliations:** 1 Myocardial Function Section, National Heart and Lung Institute, Imperial College London, 4th floor Imperial Centre for Translational and Experimental Medicine, Hammersmith Campus, Du Cane Road, London, W12 0NN UK; 2 Department of Aeronautics, Imperial College London, South Kensington Campus, London, SW7 2AZ UK; 3 Department of Cardiothoracic Transplantation & Mechanical Circulatory Support, Royal Brompton and Harefield NHS Foundation Trust, London, UB9 6JH UK; 4 ElectroCardioMaths Programme, Imperial Centre for Cardiac Engineering, Imperial College London, London, UK

## Abstract

The contact cardiac electrogram is derived from the extracellular manifestation of cellular action potentials and cell-to-cell communication. It is used to guide catheter based clinical procedures. Theoretically, the contact electrogram and the cellular action potential are directly related, and should change in conjunction with each other during arrhythmogenesis, however there is currently no methodology by which to concurrently record both electrograms and action potentials in the same preparation for direct validation of their relationships and their direct mechanistic links. We report a novel dual modality apparatus for concurrent electrogram and cellular action potential recording at a single cell level within multicellular preparations. We further demonstrate the capabilities of this system to validate the direct link between these two modalities of voltage recordings.

## Introduction

The contact electrogram (EGM) is routinely recorded in the clinical cardiac catheter laboratory during ablation procedures. It is well established that electrogram morphology is a result of the interaction of electrical activation and architecture of the local myocardium^1,2^, which has led to catheter ablation treatment strategies directly targeting areas of abnormal EGM morphology^1^. However, progress in this field has reached a bottleneck, with no recent increase in success rates due to a lack of mechanistic insight into the role of the underlying cellular and tissue level factors influencing EGM morphology. A greater understanding of this relationship is needed to more directly target treatment strategies specific to the underlying aetiology. Although previous attempts have been made to correlate cellular action potential (AP) duration and the extracellular field potential recordings^3–8^, these early methods lack either co-localisation of the action potentials and the electrogram signals^5^ or collection of high resolution data^3,6,7^. These two factors are of importance, firstly when there may be spatio-temporal heterogeneity and, secondly, for detailed cellular mechanistic insight. However, to date, no technology exists to determine these factors concurrently at single cell resolution in multicellular preparations.

Healthy myocardium gives rise to a simple EGM, with single positive and negative deflections. However, arrhythmogenesis can lead to changes in EGM characteristics rendering the morphology more complex^9^, with multiple deflections contained within each EGM. Some postulated mechanisms underlying complex EGMs include ion channel abnormalities, tortuous conduction paths through fibrotic tissue, conduction slowing, rotational activity, wavefront collision, and far-field signals^9^. With the assumption that complex fractionated EGMs represent sites of disease and areas of interest, there was a trend towards ablating these sites as an adjunctive treatment for atrial fibrillation in the last decade^10^. However, it has recently become clear that binarising EGMs into simple and complex, and then targeting of sites with complex EGMs confers no additional benefit^11^. This highlights the need to move beyond the simple binarisation of EGMs and the need, and potential benefit, of a more comprehensive understanding of the cellular basis of the contact EGM.

In terms of fundamental physics, the origin of the contact EGM is the superposition or summation of the electric field of charged ions in the vicinity of the electrode. Both the ion flux across cell membranes, producing the action potential, and the propagation of the action potential within the tissue lead to variations in electric field strength at the electrode and the subsequent EGM morphology^12^. Therefore, specific features of the EGM morphology could be used to infer the presence of, and potentially quantify, particular cellular electrophysiological factors responsible for specific EGM features, thereby providing a method to identify potential therapeutic targets more specifically. However, to date the direct relationship between characteristics of the EGM, myocardial structure and cellular and tissue level electrophysiology remains unclear, due to the lack of ability to extract meaningful causal relationships with cellular factors by simultaneously recording cellular activity alongside contact EGMs, particularly in intact preparations. The need for micro- to macro-electrophysiology extends past cardiac electrophysiology and is relevant to any electrophysiologically active organ, in particular the brain^13^.

Several methodological approaches exist for measuring electrophysiological activity in an *in vitro* or *ex vivo* setting. Commercially available research microelectrode array (MEA) systems allow recording of extracellular unipolar EGMs in *in vitro* models with a high signal to noise ratio^14^. The use of MEAs for the study of cardiac myocyte and embryonic stem cell-derived cardiomyocyte electrical activity is well established^14–16^ with recent applications on human-induced pluripotent stem cells (hiPSCs)^17^.

In contrast, optical mapping of transmembrane voltage is an electrophysiological technique used to study cellular action potentials (AP) with high spatiotemporal resolution after staining cardiac myocytes or the whole heart^18^ with voltage-sensitive dyes^19^. It gives the ability to measure both activation and repolarisation times from individual and multiple sites^19^. Optical mapping was developed as a technology to facilitate the investigation of APs in systems where the use of microelectrodes was not convenient or possible, due to scale, topology or complexity reasons^18^ and does not require complex technical skills associated with traditional electrophysiological techniques. Development of new molecular probes and advancements in optical imaging technology have increased the efficiency of optical mapping^18^. Among the technical difficulties that have been overcome over the recent years is the improved spatial and temporal resolution. For this reason, new charge-coupled device (CCD) cameras, photodiode arrays (PDA) and complementary metal-oxide semiconductor (CMOS) cameras have been developed^20–23^. CMOS technology preserves the quantum efficiency of CCD cameras, while allowing high-speed image acquisition^18^ facilitating single cell resolution and the subsequent analysis of AP heterogeneities.

Here we describe a novel non-invasive methodology which combines optical mapping at a single cell resolution within multicellular preparations with MEA recordings, for obtaining simultaneous APs and EGMs to sub-millimetre accuracy, compared to previous work^3,6,7^, using *in vitro* neonatal rat ventricular myocytes (NRVMs) and *ex vivo* human cardiac slices. We present this unique dual modality apparatus and demonstrate applications of the technique by characterisation of EGM morphology alterations due to the blockade of specific ion channels or gap junction uncoupling and how these are related to corresponding modifications of AP morphology. This provides the first direct and concurrent validation of how AP changes at a single cell level can manifest on EGM morphology on multicellular preparations.

## Results

### Synchronisation of dual modality concurrent action potential and electrogram recordings

**Figure 1:**
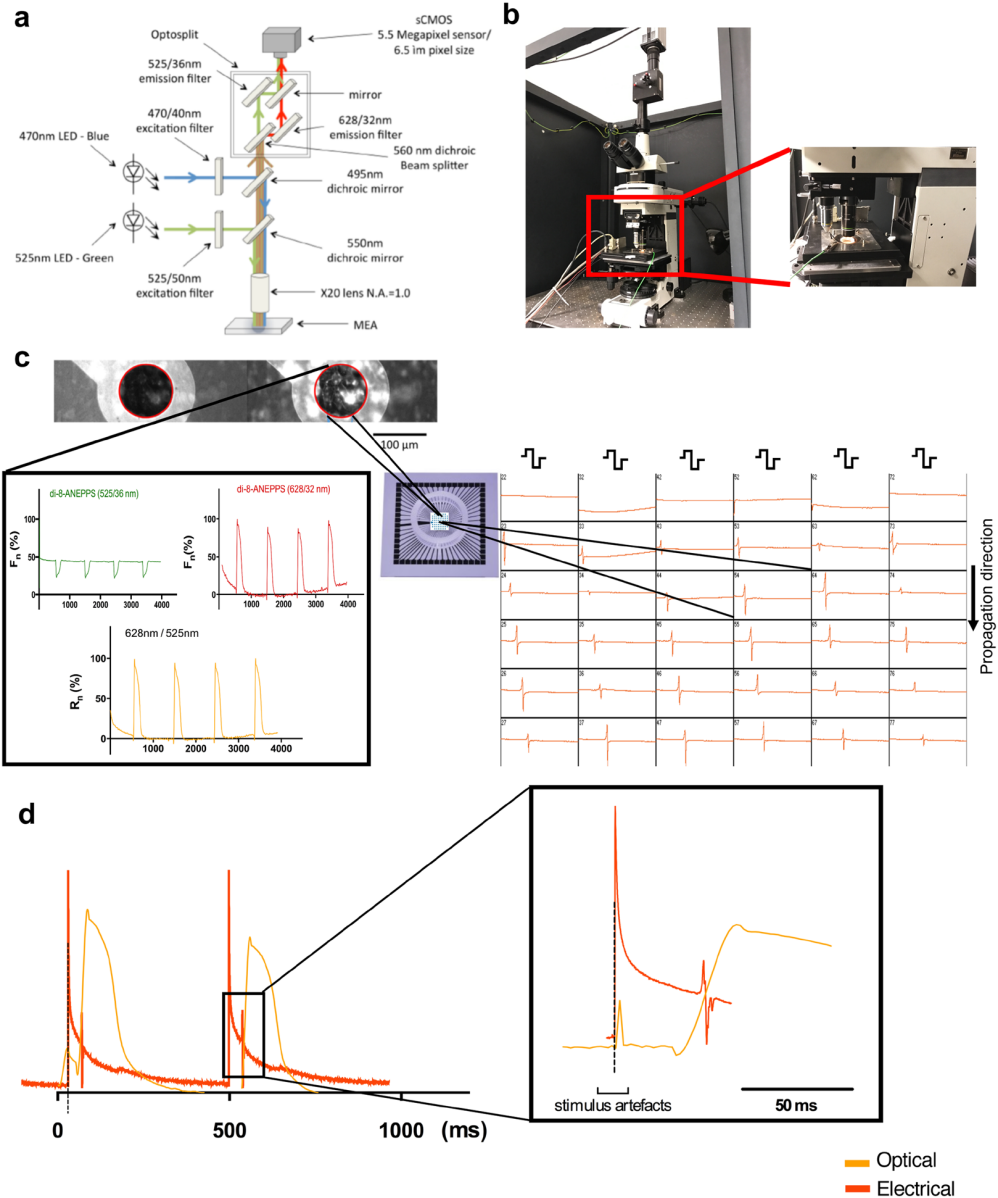
Set up for simultaneous optical mapping and electrogram recordings. (**a)** Schematic diagram of the optical mapping system connected with the MEA system for dual modality recording. (**b)** The amplifier of the MEA system is arranged on the stage of the optical system. (**c)** (Top) Image obtained through optical mapping of a NRVM monolayer stained with di-8-ANEPPS, taken over a MEA plate and as seen with simultaneous excitation of green (left) and red light (right). Outline of electrode in red circle. The change in fluorescence intensity over time on cells is used for recording the AP. (Bottom left) Signal correction by 628 nm/525 nm ratiometry (black; expressed as percentage of normalised R_n_) for simultanaeously collected fluorescence at 525/36 nm (green) and 628/32 nm (red) (both expressed as percentage change of normalised signal F_n_) emitted by the voltage sensitive fluorescent dye di-8-ANEPPS. The microelectrode array is used for recording electrograms, as presented in the screenshot (bottom right). (**d)** (Left) Representative optical and electrical signals acquired simultaneously from the same electrode with the false optical mapping stimulus artefact being synchronised with the MEA stimulus artefact. They are both connected through the dotted line. (Right) Schematic illustration of the optical action potential and unipolar EGM indicated in square to the left, where it is visible that the minimum dV/dt of the electrical signal is co-localised with the maximum optical dV/dt. The stimulus artefacts from both signals are synchronised and the dotted line confirms this.

During dual modality recordings, the MEA plate is connected to the amplifier of the MEA system and the amplifier is loaded on the stage of the custom made optical mapping system (Fig. 1). The MEA stimulator and the optical mapping camera are connected through a BNC sync cable, to provide a blank frame when the stimulator fires to give a false stimulation artefact on the optical recording for time correlation between the two recording modalities. Representative correlations between electrical and optical mapping signals and stimulus artefacts are shown in Fig. 1d. MEA recordings were performed for 10s and during this time period 5s ratiometric voltage optical mapping recordings were simultaneously obtained. Further details are described in the Methods section. Signals were recorded at the baseline sample state and after the administration of ion channel blockers or the gap junctional blocker, carbenoxolone (CBX), at IC_50_ at increasing pacing rates until loss of capture. Dual modality recordings were carried out on cardiac myocyte cultures and human ventricular myocardial slices. In addition to the entire NRVM culture, individual cells overlaying an electrode were also studied.

### Action potential upstroke can be correlated with electrogram duration and conduction velocity

**Figure 2:**
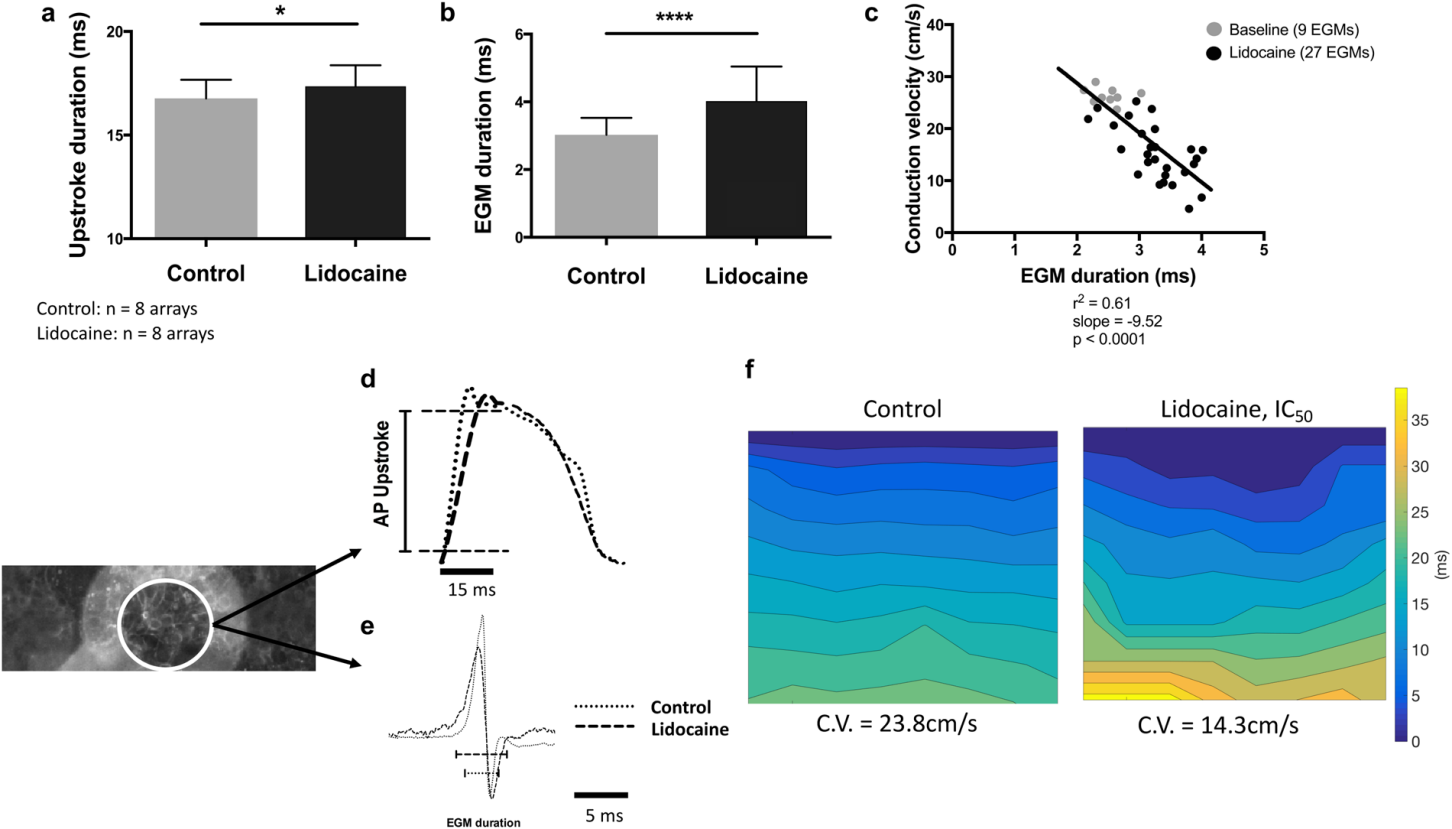
Effects of lidocaine on action potential upstroke, electrogram duration and conduction velocity in NRVM cultures. Unpaired Student’s *t*-test (two-tailed) showing the effect of lidocaine (27 μmol/L) on upstroke duration (**a)** and EGM duration (**b**) measured before or after the addition of lidocaine (n = 8). (**c)** Correlation of upstroke duration with conduction velocity before or after sodium channel blockade (r^2^ = 0.6, p < 0.0001). (**d**) Superimposed optical action potentials showing increased upstroke duration after the addition of lidocaine, and (**e**) superimposed raw unipolar EGMs, which show decreased EGM amplitude and increased EGM duration due to the blockade of sodium channels, all derived from the same electrode as shown on the left. **(f)** Isochronal maps presenting the propagation of electrical activity before (23.8 cm/s) and after (14.3 cm/s) lidocaine administration in the same NRVM culture. All bar charts represent mean ± SEM; *p < 0.05; ****p < 0.0001.

The effects of Na^+^ channel blockade on AP are reflected in the upstroke duration. Administration of the IC_50_ of lidocaine (27 μmol/L) led to an increase of both the upstroke duration (control 16.77 ± 0.9 ms, lidocaine 17.36 ± 1 ms, n = 6, p < 0.05) and the EGM duration (control 3.03 ± 0.5 ms, lidocaine 4.02 ± 1 ms, n = 6, p < 0.0001) (Fig. 2). Linear regression analysis (Fig. 2c) shows direct correlation between upstroke duration and conduction velocity (r^2^ = 0.6, p < 0.0001).

### APD_90_ can be correlated with electrogram field potential duration

**Figure 3:**
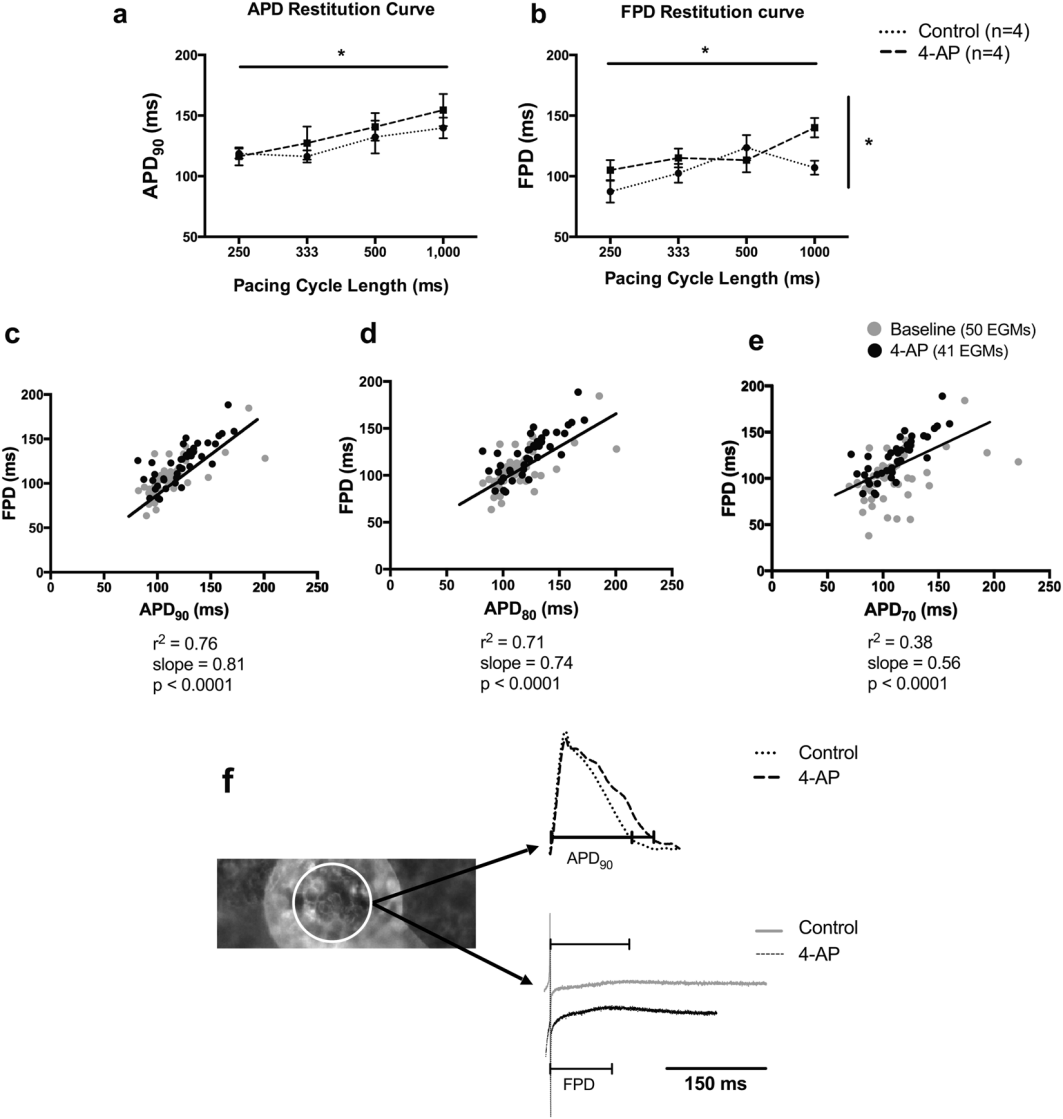
Correlations between APD_90_ and FPD in cardiac myocyte cultures following the I_to_ current blockade. Effect of 739.9 μmol/L 4-AP on APD_90_ **(a)** and FPD **(b)** (Control: n = 4; 4-AP: n = 4). APD_90_ and FPD differed significantly both among pacing rates (p < 0.05), but only FPD was affected by I_to_ blockade (p < 0.05, ordinary two-way ANOVA). Linear regression correlating APD_90_ **(c)**, APD_80_ **(d)** and APD_70_ **(e)** with FPD due to the IC_50_ concentration activity of 4-AP (APD_90_: r^2^ = 0.76, slope = 0.81; APD_80_: r^2^ = 0.71, slope = 0.74; APD_70_: r^2^ = 0.38, slope = 0.56; p < 0.0001). (**f)** Superimposed raw traces obtained before and after gap junction blockade by carbenoxolone and the concurrent EGMs. The raw traces were obtained from the same electrode as indicated on the left. Mean ± SEM; ***p < 0.001; ****p < 0.0001.

The blockade of the transient outward potassium (I_to_) current, using 4-aminopyridine (4-AP), affected both action potential duration (APD) and EGM field potential duration (FPD), the time between depolarisation and repolarisation (Fig. 3). Electrical restitution occured when stimulating cells at progressively reduced pacing intervals from 1000 to 250 ms both in control state and after I_to_ blockade using the IC_50_ of 4-AP (739.9 μmol/L) (Fig. 3a and b). Rate adaptation of APD to 90% repolarisation (ΑPD_90_) was affected by pacing rate (control: 1 Hz 139.7 ± 19.2 ms, 4 Hz 118.7 ± 9.4 ms, n = 4; 4-AP: 1 Hz 154.6 ± 29.7 ms, 4 Hz 116.3 ± 14.7 ms, n = 4; p = 0.04). FPD was affected both by pacing rate (p = 0.04) and I_to_ blockade (p = 0.02) (control: 1 Hz 107.09 ± 12.9 ms, 4 Hz 87.3 ± 20.3 ms, n = 4; 4-AP: 1 Hz 140 ± 18 ms, 4 Hz 105 ± 16.6 ms, n = 4), but without interaction between the variables. There was a strong correlation between APD_90_ and FPD (r^2^ = 0.76, slope = 0.81, p < 0.0001) as measured by linear regression (Fig. 3c). Although there was correlation between APD_80_ and FPD (r^2^ = 0.71, slope = 0.74, p < 0.0001) and APD_70_ and FPD (r^2^ = 0.38, slope = 0.56, p < 0.0001), correlation slopes were shallower than for APD_90_ (Fig. 3d and e). The relationship between APD_90_/FPD remained the same before and after 4-AP administration, demonstrated by no significant difference between the correlation coefficients and slopes before and after I_to_-current blockade groups (control: r^2^ = 0.5, slope = 0.63; 4-AP: r^2^ = 0.62, slope = 0.83; p = 0.18).

**Figure 4:**
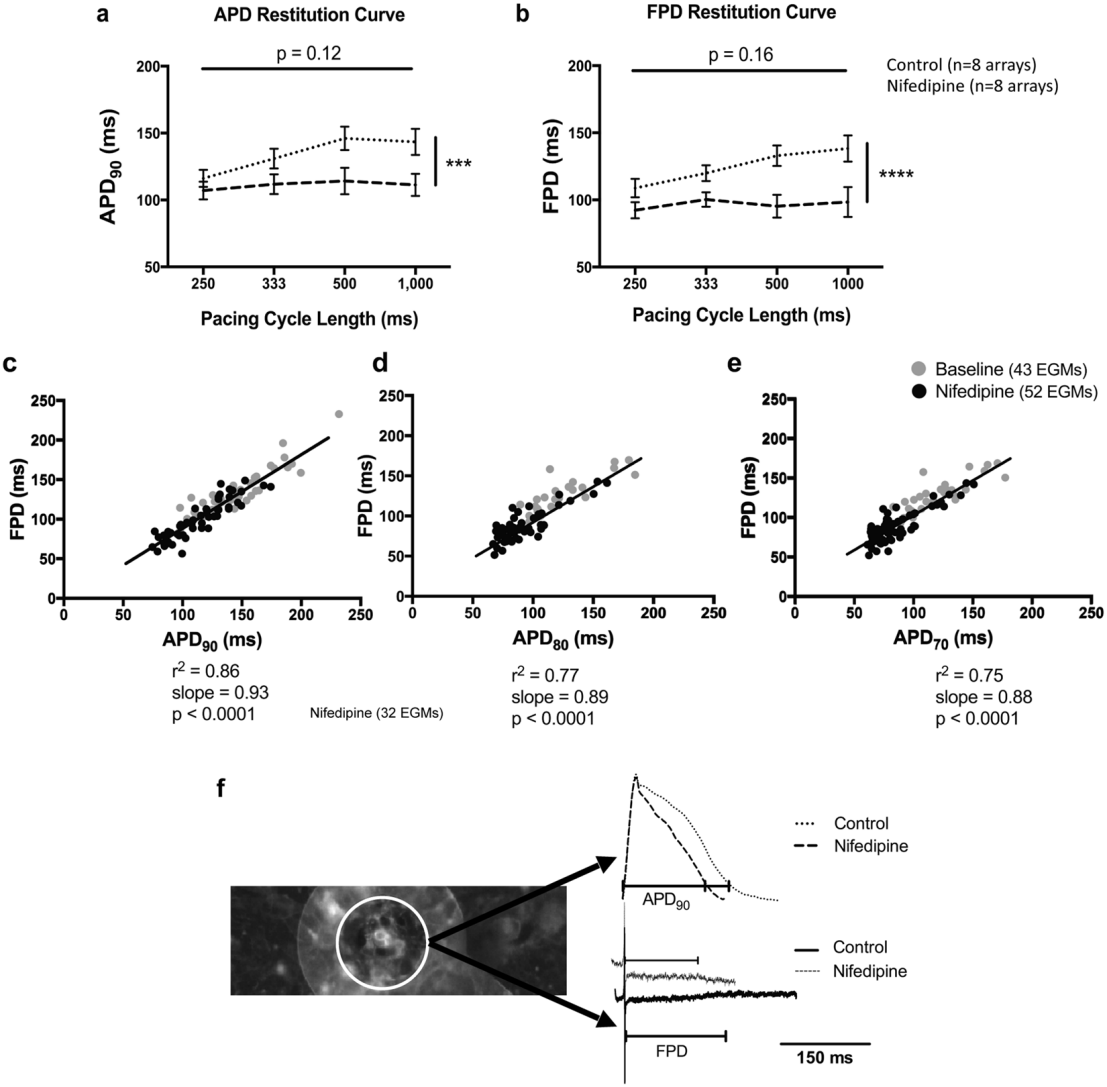
Correlations between APD_90_ and FPD in cardiac myocyte cultures after the L-type Ca^2+^ channel blockade. APD_90_ (**a**) and FPD (**b**) restitution relations before and after the application of 772nmol/L nifedipine (n = 8 for each group of data). APD_90_ and FPD differed significantly before and after the treatment with nifedipine (p < 0.001 and p < 0.0001 respectively, ordinary two-way ANOVA). (**c)** The linear relationship between APD_90_ and FPD (r^2^ = 0.86, slope = 0.93, p < 0.0001). Linear regression analysis for the relationship between **(d)** APD_80_ (r^2^ = 0.77, slope = 0.89, p < 0.0001) or **(e)** APD_70_ (r^2^ = 0.75, slope = 0.88, p < 0.0001) and FPD. (**f)** Superimposed raw traces obtained before and after suppression of I_CaL_ by nifedipine and the concurrent EGMs, all derived from the same electrode on the left. Mean ± SEM; ***p < 0.001; ****p < 0.0001.

APD was found to be dependent on both L-type Ca^2+^ channel activity and cycle length. Rate adaption of APD_90_ and FPD, before the blockade of L-type Ca^2+^ channels by the IC_50_ of nifedipine (772 nmol/L), was seen. Both APD_90_ (control: 1 Hz 143.44 ± 29 ms, 4 Hz 116.13 ± 18 ms; nifedipine: 1 Hz 111.3 ± 23 ms, 4 Hz 107 ± 19 ms, n = 8, p < 0.001) and FPD (control: 1 Hz 138.3 ± 29 ms, 4 Hz 108.8 ± 19 ms; nifedipine: 1 Hz 98.5 ± 31 ms, 4 Hz 92.4 ± 17 ms, n = 8, p < 0.0001) decreased due to I_CaL_ blockade, but remained constant with respect to cycle length (Fig. 4a,b). Linear regression analysis (Fig. 4c) showed that APD_90_ and FPD are strongly correlated (r^2^ = 0.86, slope = 0.93, p < 0.0001). The APD_90_/FPD correlation, as calculated using the data before and after nifedipine activity, was not significantly different between the two groups (Control: r^2^ = 0.84, slope = 0.74; nifedipine: r^2^ = 0.81, slope = 0.75; p = 0.65). There is also a direct correlation, even though with a shallower slope, between APD_80_ and FPD (Fig. 4d: r^2^ = 0.77, slope = 0.89, p < 0.0001) and APD_70_ and FPD (Fig. 4e: r^2^ = 0.75, slope = 0.88, p < 0.0001).

**Figure 5:**
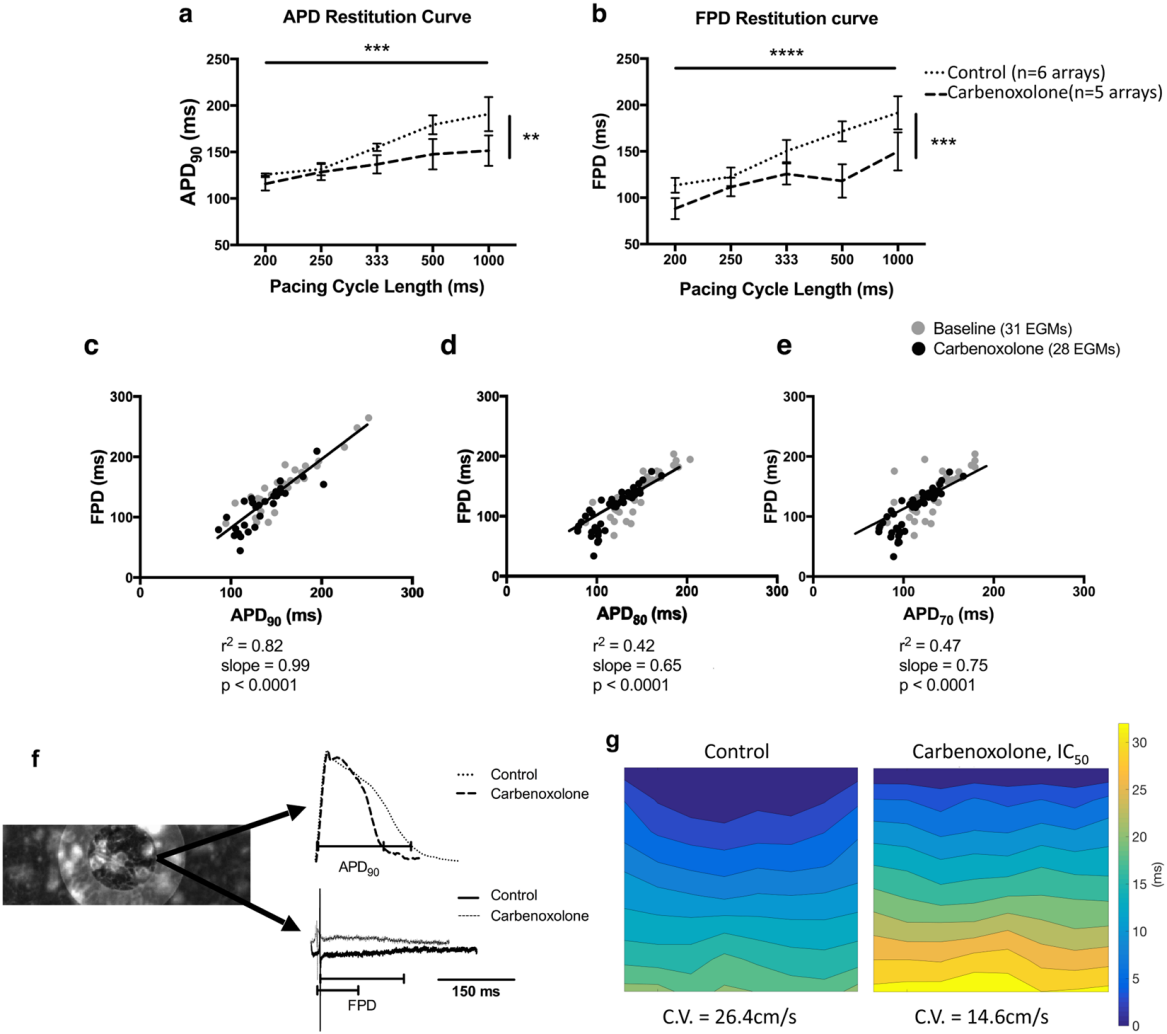
Correlations between APD_90_ and FPD in cardiac myocyte cultures after the gap junction uncoupling. Effect of 15.8 μmol/L CBX on APD_90_ (**a**) and FPD (**b**) (Control: n = 6; CBX: n = 5). APD_90_ and FPD differed significantly both among pacing rates (p < 0.001 and p < 0.0001 respectively) and due to gap junction uncoupling (p < 0.01 and p < 0.001 respectively, ordinary two-way ANOVA). Linear regression correlating APD_90_ **(c)**, APD_80_ **(d)** and APD_70_ **(e)** with FPD due to gap junction uncoupling by CBX (APD_90_: r^2^ = 0.847, slope = 0.99; APD_80_: r^2^ = 0.42, slope = 0.65; APD_70_: r^2^ = 0.47, slope = 0.75; p < 0.0001). (**f)** Superimposed raw traces obtained before and after gap junction blockade by CBX and the concurrent EGMs. The raw traces were obtained from the same electrode as indicated on the left. **(g)** Isochronal maps presenting the propagation of electrical activity before (26.4 cm/s) and after (14.6 cm/s) gap junction uncoupling in the same NRVM culture. All bar charts represent mean ± SEM; ***p < 0.001; ****p < 0.0001.

Gap junctional block was observed to affect APD. Electrical restitution occured when stimulating cells at progressively reduced pacing intervals from 1000 to 200 ms both in control state and after gap junction uncoupling using the IC_50_ of carbenoxolone (CBX, 15.8 μmol/L) (Fig. 5a and b). Both APD_90_ (control: 1 Hz 190.78 ± 48 ms, 4 Hz 125.7 ± 3 ms, n = 6; CBX: 1 Hz 151.5 ± 37 ms, 4 Hz 115.7 ± 17 ms, n = 5; p < 0.01) and FPD (control: 1 Hz 191.5 ± 47 ms, 4 Hz 113.4 ± 18 ms, n = 6; CBX: 1 Hz 149.8 ± 46 ms, 4 Hz 88.3 ± 27 ms, n = 5; p < 0.001) were affected by pacing frequency and CBX activity. Linear regression analysis revealed the presence of a direct correlation between APD_90_ and FPD (r^2^ = 0.847, p < 0.0001) with CBX (Fig. 5c). There was no significant difference in correlation coefficients and slopes between the before and after gap junction uncoupling data groups (Control: r^2^ = 0.85, slope = 0.9; CBX: r^2^ = 0.72, slope = 0.83; p = 0.86). There was correlation with a shallower slope for APD_80_ and FPD (r^2^ = 0.42, slope = 0.65, p < 0.0001) and APD_70_ and FPD (r^2^ = 0.47, slope = 0.75, p < 0.0001) (Fig. 5d,e) compared to APD_90_. The mean conduction velocity before and after the addition of CBX on NRVM cultures was 20.6 ± 1.3 cm/s and 11.5 ± 5.8 cm/s respectively (Control: n = 6 arrays; CBX: n = 5 arrays; p < 0.001).

### Single cell level correlations of action potential timings and electrogram fractionation

**Figure 6:**
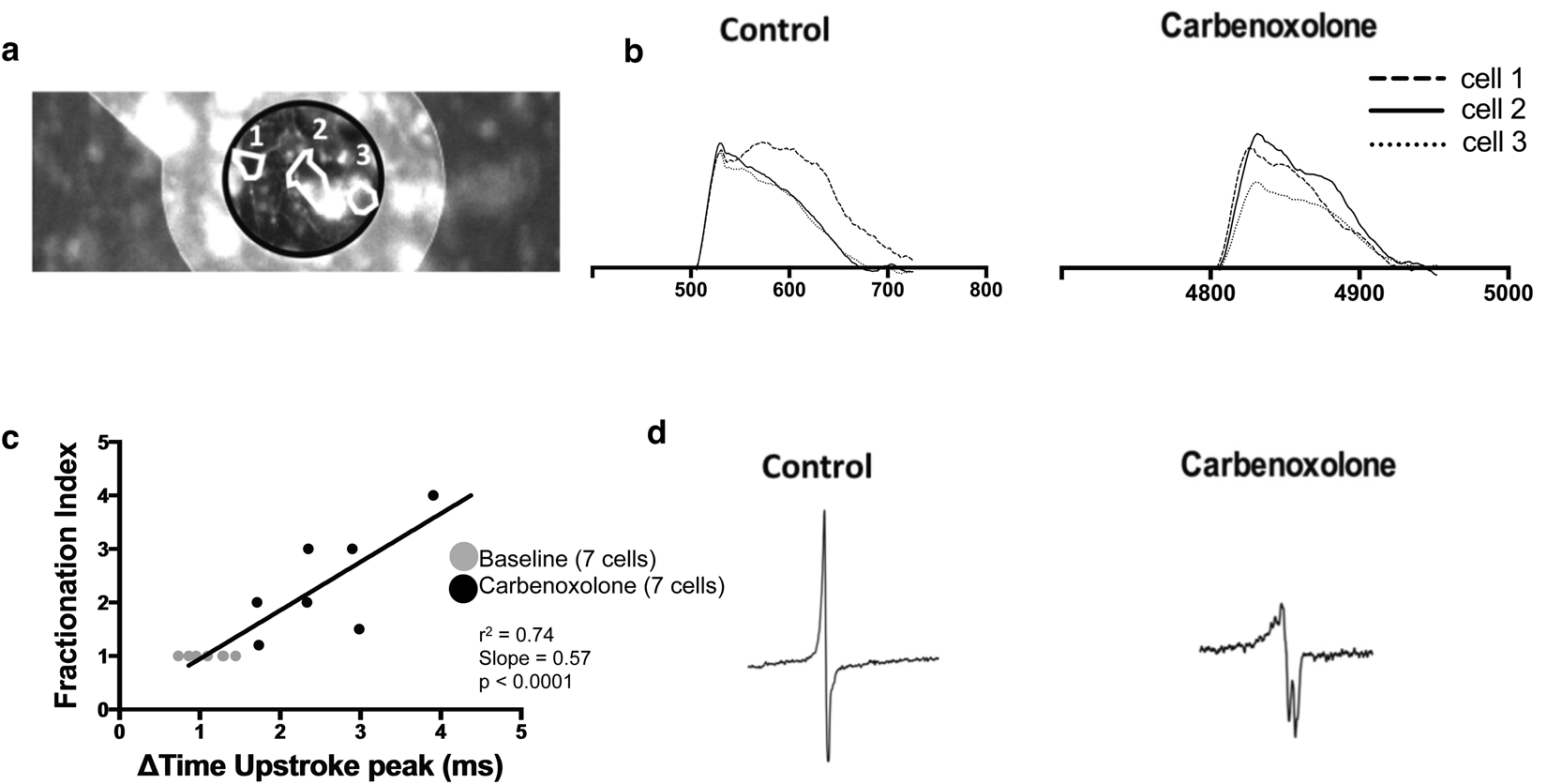
Correlation of action potential timing heterogeneity and EGM fractionation at the single cell level. **(a)** Image of a NRVM monolayer obtained through optical mapping. Outline of electrode (black circle) and three single cells (white outline) located across the electrode. (**b)** Superimposed optical action potentials derived from three single myocytes either at the baseline state or after the effect of CBX. Cells were located across an electrode (as in 5a). Upstroke duration is the same for control cells (left), but the spread increases alongside the AP propagation after gap junction uncoupling (right). (**c)** Correlation of the time change of upstroke peaks between cell positions 1 and 3 with the level of EGM fractionation before and after the addition of 15.8 μmol/L CBX on NRVM (r^2^ = 0.74, p < 0.0001). (**d)** EGMs obtained before (left) and after (right) the addition of CBX showing decreased EGM amplitude, increased fractionation and longer EGM duration as consequences of gap junction uncoupling.

To determine whether gap junction uncoupling affects both AP upstroke timing heterogeneity and EGM fractionation, dual modality recordings before and after the addition of 15.8 μmol/L CBX (IC_50_) were analysed for individual myocytes coming from the same group of cells on top of an electrode. The AP traces, obtained from single cells before and after the addition of CBX, showed that the time point of upstroke peak is similar at baseline, but showed a larger distribution range across an electrode after gap junction uncoupling (Fig. 6b). CBX administration also led to increased EGM fractionation (Fig. 6d). Linear regression analysis shows that the time difference (ΔT) between the upstroke peak time points of two cells located at equal points across an electrode is directly correlated with EGM fractionation (r^2^ = 0.74, slope = 0.57, p < 0.0001, n = 14) (Fig. 6c).

### Correlation between APD_90_ and FPD remains in intact human ventricular slices

**Figure 7:**
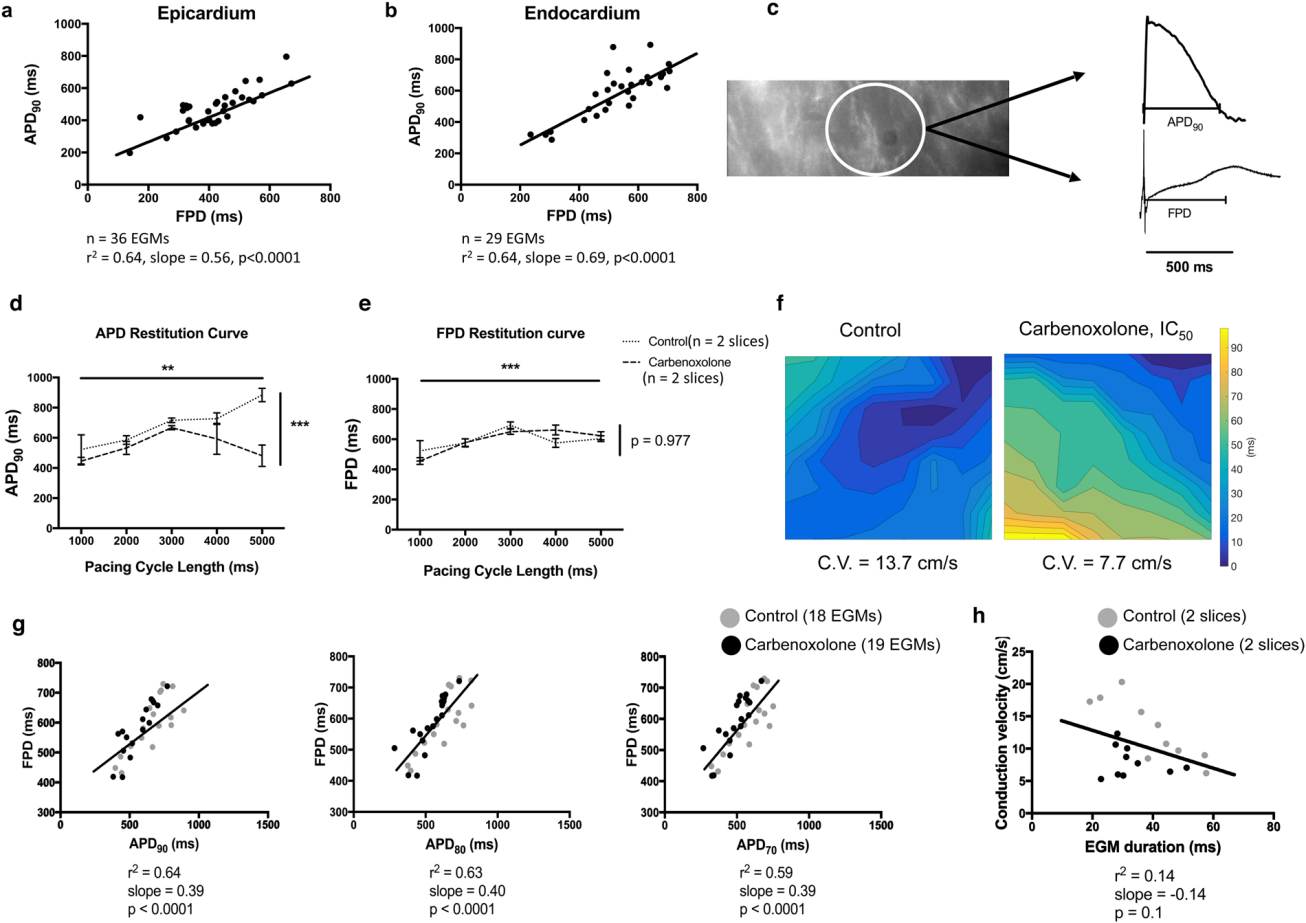
Correlation between APD_90_ and FPD in human ventricular slices. (**a,b)** MEA and optical mapping recordings obtained simultaneously from human heart slices derived from the left ventricular epicardium (n = 36 EGMs, obtained from 8 slices in total) and endocardium (n = 29 EGMs, obtained from 6 slices in total) show that APD_90_ and FPD are strongly correlated in both sites (r^2^ = 0.64, p < 0.0001 for both cases). (**c)** Raw trace obtained during optical mapping from human epicardial ventricular slice and the concurrent EGM, as both derived from the same electrode presented on the left. It is highlighted that APD_90_ and FPD are similar (500 ms). Effect of 15.8 μmol/L CBX on APD_90_ (**d**) and FPD (**e**) (Control: n = 2; Carbenoxolone: n = 2). APD_90_ and FPD differed significantly both among pacing rates (p < 0.01 and p < 0.001 respectively) and APD_90_ only changed significantly due to gap junction uncoupling (p < 0.001, ordinary two-way ANOVA). **(f)** Isochronal maps presenting the AP propagation before (14.2 cm/s) and after (9.2 cm/s) gap junction uncoupling in adjacent tissue slices. The markedly slow conduction in the centre of the control slice is due to the presence of a fibrotic area within the slice preparation. **(g)** Linear regression correlating APD_90_, APD_80_ and APD_70_ with FPD using data before and after the CBX activity on neighbouring heart failure tissue slices (APD_90_: r^2^ = 0.64, slope = 0.39; APD_80_: r^2^ = 0.63, slope = 0.4; APD_70_: r^2^ = 0.59, slope = 0.39; p < 0.0001). All bar charts represent mean ± SEM; **p < 0.01; ***p < 0.001. **(h)** Linear regression analysis correlating conduction velocity measured before and after gap junction uncoupling with the EGM duration of the data obtained under the same conditions (r^2^ = 0.14, slope = −0.14, p = 0.1).

Concurrent MEA and optical mapping recordings were obtained from *ex vivo* adult human cardiac slices derived from the epicardial (n = 8) or the endocardial (n = 6) left ventricular wall. Slices were stimulated from 0.2 Hz to 1 Hz. These intact slice results reconfirm those of the cell monolayer models; there is a direct correlation between APD_90_ and FPD, both in the epicardial and the endocardial samples (r^2^ = 0.64, p < 0.0001) (Fig. 7). Futhermore, our study also shows that gap junction uncoupling modified both AP and EGM morophology. An indicator of the effect of CBX on tissue slices is the reduction in conduction velocity from 11.85 ± 3.3 cm/s (n = 2 slices) to 6.9 ± 3.2 cm/s (n = 2 slices) using spatially serial tissue slices (Fig. 7f). APD_90_ changed due to gap junction uncoupling (control: 0.2 Hz 883.6 ± 77 ms, 1 Hz 522.6 ± 194.6 ms; CBX: 0.2 Hz 481.3 ± 122.8 ms, 1 Hz 445.5 ± 50.6 ms; n = 8; p < 0.001), and restitution was seen due to pacing frequency (p < 0.01). The interaction effects between gap junction uncoupling and pacing frequency were statistically significant (p < 0.05, ordinary two-way ANOVA) (Fig. 7d). FPD was only affected by pacing rate (p < 0.001) and not by CBX activity (control: 0.2 Hz 603.9 ± 33.2 ms, 1 Hz 522.5 ± 135.1 ms; CBX: 0.2 Hz 622.5 ± 46.3 ms, 1 Hz 455.7 ± 45 ms; n = 4) (Fig. 7e). There is linear correlation between APD_90_ and FPD (r^2^ = 0.64, slope = 0.39, p < 0.0001) measured using the before and after gap junction uncoupling dual modality data. The APD_90_/FPD correlation was not significantly different between the two groups (Control: r^2^ = 0.63, slope = 0.51; CBX: r^2^ = 0.77, slope = 0.68; p = 0.24). However, there was correlation with a shallower slope between APD_80_ and FPD (r^2^ = 0.63, slope = 0.4, p < 0.0001) and APD_70_ and FPD (r^2^ = 0.59, slope = 0.39, p < 0.0001) (Fig. 7g) compared to APD_90_. When correlating conduction velocities with EGM duration, although there was a qualitative increase in EGM duration with a decrease in conduction velocity, this did not reach statistical significance (r^2^ = 0.14, slope = −0.14, p = 0.1) (Fig. 7h). Fractionation of EGMs was observed before CBX administration (2.6 ± 0.2, n = 26 EGMs) and increased slightly after CBX administration (3 ± 0.2, n = 27 EGMs), but this change was not statistically significant (p = 0.195).

## Discussion

We present the first application of a unique technique, adequate for identifying single cells within multicellular preparations, for the simultaneous recording of contact EGMs and optical action potentials in 2D preparations providing direct mechanistic insight into how changes in AP morphology directly manifest in unipolar EGM morphology. Our novel method enabled us to obtain high resolution data using electrodes which have an order of magnitude smaller diameter and intra-electrode distance less than half of that presented on previous systems^6,7^. We further demonstrate application of this novel technique using both cell monolayer and intact tissue slice models. Although EGM correlates of AP changes have previously been characterised^3–8^, this methodology allows direct and comprehensive analyses of all factors affecting AP morphology and its propagation, and allows concurrently recorded consequent EGMs to be determined. The simplicity of the MEA and optical mapping techniques allows these electrophysiological analyses to be carried out without the need for specific expertise in complex electrophysiological techniques such as patch clamp^3,4,24^. Moreover, the technique that we present here allows the simple colocalisation of optical mapping and electrogram recordings, and as a result it can be easily applied in a biological lab with relatively low cost, compared to previously published devices^8,25^, making it more accessible. This system allows analyses of summated EGMs alongside the cumulative single cell APs contributing to that EGM, allowing for investigation of how cellular level heterogeneities impact EGM morphology.

Because of the clinical importance of contact EGMs^26^ in both the diagnosis and treatment of arrhythmias, there is a strong interest in associating abnormalities in the EGM morphology with modifications occurring at the tissue and cellular levels and numerous studies have attempted to clarify this^27–29^, however these findings are each in isolation and there is a lack of comprehensive mechanistic understanding. The use of this novel dual modality system to comprehensively characterise the direct and concurrent relationships between EGMs and APs will provide added value to future studies where only non-invasive EGMs can be recorded. As proof of concept we modulated a number of AP characteristics to determine their impact on the EGM.

In all preparations a delay between the stimulus artefact and response was observed. The stimulation voltage was kept at 120% of the threshold voltage, which is low enough so as not to damage the preparations with excess energy, however, as a result, cell response is delayed both in EGMs and action potentials. Delayed initial depolarisation when using low voltage stimulation was first described by Hodgkin and Huxley^30^. In addition to this, when recording from an electrode which is located at a distance from the stimulus electrode, the time taken for propagation to occur through the preparation needs to be taken into account. The above phenomena together can explain the delay between stimulation artefact and recorded action potential/EGM initiation.

We have demonstrated that lidocaine, a class 1B antiarrythmic agent and Na^+^-channel blocker, led to conduction delay in NRVMs, which is in general agreement with previous studies^31^. Our dual modality data show both prolonged upstroke and EGM duration concurrently in the same cells, providing evidence of a mechanistic link. Longer upstroke duration due to I_Na_ blockade leads to slower conduction velocity and since EGM duration is the time needed for the activation wavefront to pass across an electrode, slower conduction velocity leads to longer EGM duration. This phenomenon was also observed in the human slices, though to a lesser extent, because the rapid upstroke showed little variation within the temporal resolution of the mapping system.

HERG K^+^ current and ventricular I_to_ are blocked by 4-AP^32,33^. This compound was used to block I_to_ in NRVM cultures and subsequently modulated APD_90_. Although APD_80_ and APD_70_ were also increased due to 4-AP activity, their correlation with FPD was weaker than for APD_90_. FPD has previously been described as an EGM feature on MEAs, defined as the time between the depolarisation peak (minimum voltage) and the repolarisation peak (maximum voltage), and is thought to be an index of APD_90_^34^. Not only this I_to_ current modulation, but all functional modulations investigated in this study validated that APD_90_ is best correlate of FPD and this relationship is retained with modulation of APD_90_.

Nifedipine is well studied for its effects on L-type Ca^2+^-channel blockade^35,36^, and was used here to modulate APD_90_. The simultaneous MEA and optical mapping recording showed that FPD can be used as a predictor of APD_90_, since they are both reduced simultaneously on the administration of nifedipine. A direct relationship between FPD and APD_90_ is also demonstrated with CBX administration. CBX, a gap junction blocker, has been studied for its effects on conduction slowing and EGM fractionation^37–39^, but it still remains unclear how it affects AP morphology. It has been suggested that gap junction uncoupling by CBX can either lead to prolonged APD in perfused adult rat hearts^40^ or it has no effect on the APD of rabbit ventricular myocytes^38,39^. However, the AP data we collected concurrently with EGM recordings confirmed a direct correlation between FPD and APD_90_. Therefore we have validated the use of our technique in correlations of action potential depolarisation, repolarisation and propagation, demonstrating the capabilites to investigate all aspects of cellular electrophysiology.

Recently, intact myocardial slices are increasingly being used, due to the retention of structural and functional properties of the native myocardium^34^ compared to *in vitro* preparations. There have been attempts in the past to measure the EGMs on myocardial slices derived from human, guinea pig or rabbit heart^34,41^. However, this study demonstrated the capabilites of the dual modality system to record concurrent APs and MEAs from the same area of human heart failure slices, in order to correlate EGM and AP morphology modifications. We obtained data from endocardial and epicardial left ventricular slices showing that there is a direct correlation between FPD and APD_90_. The dual modality results corroborated that this relationship was present even after modulation of gap junction coupling using CBX on epicardial slices. This further validated the results obtained from the *in vitro* model in a more complex *ex vivo* intact tissue model and demonstrated the translational benefit of this method. The activation maps acquired from the slice preparation show non-uniform propagation, even during baseline conditions. This may be due to the line of stimulus not being parallel to the fibre orientation, or due to the presence of scar, as the source of human slices was patients with heart failure. However, conduction velocities were calculated as average inter-electrode velocities in the direction of propagation, and therefore, are still an accurate marker of conduction velocity in these preparations. Since MEA recordings are currently applied on brain slice preparations^42^ and the necessity of micro- to macro-electrophysiology techniques for measuring the neuronal activity have previously been justified^13^, the application of our set up for simultaneous optical imaging and MEA recordings on heart slices indicates that it could be also used for studying the neuronal activity on brain slices.

We have demonstrated the potential capability of our technique to elicit correlations between EGM and AP morphological charateristics, not only in groups of cells subjected to pharmacological manipulation, but also at the level of action potentials of individual cells located on top of an electrode, for the first time. This was possible due to the fact that the Zyla sCMOS camera, used for optical mapping of our NRVM preparations, incorporates complementary metal oxide semiconductor technology that is proved to be superior to other detector technologies in terms of spatiotemporal resolution because of the high pixel density and fast data-streaming rates^43^. Concurrently recorded AP and EGM morphology were studied before and after the administration of CBX on individual myocytes, in order to investigate the morphology alterations due to gap junction uncoupling. We showed an increase in the spread of the AP upstroke timings in cells located across an electrode due to CBX administration. These results agree with previous *in vitro* studies which show that gap junction uncoupling leads to intracellular activation delays and multiphasic optically recorded AP upstrokes^44,45^. The delay shown in our study correlated with the generation of fractionated EGMs, while no fractionated EGMs were recorded before the administration of CBX. This observation may be explained by the discontinuity in AP propagation^38^ associated with gap-junctional uncoupling. Our correlation of ΔT of action potential upstroke and EGM fractionation index suggests that in these paced preparations with linear propagating wavefronts, discontinuous propagation due to cellular uncoupling is sufficient to lead to EGM fractionation, and they can be directly correlated.

We have demonstrated this novel methodology and its possible outcomes using *in vitro* cell and slice models. However, with the availablity of flexible electrode arrays (both research and clinical) and whole heart optical mapping, it should be possible to apply our described methods on *ex vivo* whole heart models to gain insights into the cellular mechanisms leading to abnormal electrograms including the complexities of 3D structure and far-field signals.

In conclusion, we have demonstrated the utililty of a novel method combining optical mapping with MEA recordings for simultaneous recording of action potentials and EGMs with single cell resolution from the same group of cells. This technique has a number of applications in investigating electrophysiological abnormalities, as demonstrated using *in vitro* and *ex vivo* models, and can give a unique insight into cellular mechanisms leading to EGM changes. A variety of functional modulations led to different EGM changes and showed strong correlations with the corresponding AP morphology characteristic, indicating that EGM features could be used as predictors for specific changes on AP morphology from the single cell to the tissue level.

## Methods

### Cell Cultures

All reagents were sourced from Sigma Aldrich (St Louis, USA) unless otherwise stated. NRVMs were obtained from Sprague-Dawley rats (0 to 3 days old). All procedures were conducted according to the standards set by the EU Directive 2010/63/EU and were approved by the Imperial College London Ethical Committee. Anaesthesia by isoflurane was followed by euthanasia carried out by cervical dislocation, and assessed by cessation of circulation after which the heart was removed and dissected immediately. The vessels and atria were removed to isolate the ventricles which were further dissected into 1–2 mm^3^ pieces. Enzymatic digestion of ventricles was carried out using the gentleMACS neonatal heart dissociation kit (Miltenyi Biotec GmbH). The tissue and enzyme mix was incubated three times at 37 °C for 15 minutes each time before being attached to the gentleMACS dissociator for gentle agitation. The digested sample was resuspended in 10.5 mL M199 10% cell culture medium (100 mL M199, 10 mL neonate serum, 10 μM/mL penicillin-streptomycin, 0.68 mM L-glutamine, 2 μg/mL vitamin B_12_), then passed through a 70 μm pre-separation filter and centrifuged at 1000 rpm for 5 min. The cell pellet was re-suspended in 20 mL M199 10% culture medium and fibroblasts were removed by preplating cells for 1 hour at 37 °C/1% CO_2_. The remaining suspended cell population consisting of NRVMs only was extracted after a final filtration through a 70 μm pre-separation filter. 200, 000 cells were plated on microelectrode array (MEA) dishes (MultiChannel Systems MCS GmbH, Germany) coated with 20 μL 0.2 mg/mL collagen over the electrode matrix. Cultures were incubated at 37 °C/1% CO_2_ in M199 10% for the first 24 hours and then in M199 5% and measurements were recorded between the third and fourth day in culture.

### Collection and preparation of tissue samples

Human left ventricular transmural tissue samples were prepared from end-stage heart failure patients. The mean age of the patients was 57 ± 10.5 years. All samples were immersed in ice-cold cardioplegia solution (Martindale Pharmaceuticals, UK) immediately after explantation and transported to the laboratory within one hour. Samples with an approximately 6 × 6 mm surface area were obtained and mounted with epicardium down onto the specimen holder of a high precision vibrating microtome (7000 smz, Campden Instruments Ltd., UK). Samples were in cold (4 °C) oxygenated (100% O_2_) cutting Tyrode solution (140 mM NaCl, 6 mM KCl, 10 mM glucose, 10 mM HEPES, 1 mM MgCl_2_, 1.8 mM CaCl_2_, 3 g/L 2,3-butanedione monoxime (BDM), pH 7.4) during the preparation of 300 μm slices derived from the endocardial and epicardial sample areas. The advancement speed of the steel blade was 0.03 mm/s, the amplitude 2 mm and the vibration frequency was 80 Hz. Slices were incubated in oxygenated ice-cold cutting Tyrode solution for at least 30 minutes prior to being placed in the centre of a MEA plate and obtaining the electrophysiological recordings in Tyrodes solution (140 mM NaCl, 4.5 mM KCl, 10 mM glucose, 10 mM HEPES, 1 mM MgCl_2_, 1.8 mM CaCl_2_, 1 g/L BDM). In order to study the effects of gap-junction uncoupling on tissue slices, 15.83 μmol/L carbenoxolone was added in the same recording Tyrodes solution. This study was supported by the supply of human tissue samples from the Cardiovascular Research Centre Biobank at the Royal Brompton and Harefield NHS Foundation Trust (NRES ethics number for biobank samples: 09/H0504/104 + 5; Biobank approval number: NP001-06-2015). Informed consent was obtained from each patient involved in this study. All procedures described in this manuscript were carried out in accordance to the Human Tissue Act 2004 (c30).

### Microelectrode array recordings

The electrophysiological properties of cardiac myocytes were assessed using the USB-MEA60-Inv MEA system (MultiChannel Systems, Reutlingen, Germany). An MEA plate consists of 60 gold electrodes arranged on an 8 × 8 matrix (inter-electrode space: 700 μm, electrode diameter: 100 μm) with missing electrodes in the corners of the matrix. Stimulation was carried out using a STG stimulus generator programmed by MC Stimulus II software (version 3.4.4, MultiChannel Systems). A biphasic stimulus (2 ms duration; 120% of the threshold; voltage: 500–1000 mV) was applied for several minutes from the 6 electrodes located on one of the four external rows of the matrix to reach steady state before obtaining 10 sec recordings in incremental rates until loss of 1:1 capture. The temperature was kept at 37 °C during stimulation. The signals were recorded at a sampling frequency of 25 kHz. The responsiveness of cells to lidocaine (1–100 μmol/L), 4-aminopyridine (0.5–1.5 mmol/L), nifedipine (0.03–10 μmol/L) and carbenoxolone (5–100 μmol/L) dissolved in Hank’s buffered saline solution (HBSS) supplemented with 1 mM MgCl_2_ and 1.5 mM CaCl_2_ was tested 3–4 days after plating cells on MEA dishes. All chemical compounds were sourced by Sigma-Aldrich. Control recordings with NRVMs in 1 mL of HBSS were obtained before changing the control solution for 1 mL of incremental concentrations of each pharmacological agent. Signals were displayed and data were analysed offline using MC Rack software (v4.6.2, MultiChannel Systems). No filtering occurred during the post-processing analysis.

EGMs were manually annotated to extracted QRS complex duration and extracellular field potential duration (FPD). FPD was defined as the time elapsed between the initial deflection of the field potential and the maximal point of the T wave.

### Optical Mapping

Optical mapping was carried out in combination with MEA recordings. Figure 1 is a schematic representation of the custom-made optical system used for simultaneous membrane voltage and MEA recordings (supplied by Cairn Research, UK). The system was built around an upright microscope (Eclipse FN1, Nikon Instruments Europe B.V.) with a modified stage height to hold the amplifier of the MEA system. Excitation light (470 nm) was supplied by an OptoLED system (Cairn Research, UK) which provided controlled illumination and modulation. The light was passed through an emission filter of 470/40 nm. Light was collimated on the MEA dish by a water dipping objective lens with magnification 20× (NA: 1.0 20×, XLUMPLFLN20XW PL FLUORITE OBJ, Olympus). Samples were stained with 40 μM di-8-ANNEPS (Molecular Probes®, Invitrogen) diluted in 1 mL HBSS with 2.5 μL Pluronic® F-127 (Life Technologies, USA). The emitted fluorescence was collected by the same objective lens and passed through dichroic mirrors. Using a 560 nm edge BrightLine® single-edge dichroic beamsplitter, located in an Optosplit II ‘LS’ emission image splitter (x1.0 magnification), the fluorescent light was divided into two beams that were passed through emission filters (525/36 nm and 628/32 nm). Subsequently, the light was focused onto a complementary metal-oxide semiconductor camera (Zyla 10-tap sCMOS, Andor Technologies Ltd., Belfast, UK) for the detection of the dual wavelength optical signal with a spatial resolution of 400 × 885 pixels at 525.39 frames/sec. The light intensity measurements were recorded using the Andor Solis software platform (version 4.23.30008, Andor Technologies Ltd.). Optical mapping data were spatially smoothed on GraphPad Prism (version 6.0 f for Mac OS X, GraphPad Software, La Jolla California, USA) using 3 averaging neighbours and 4^th^ order of the smoothing polynomial. Action potential data were undertaken with emission ratiometry^46^. The ratiometric signal analysis was achieved by using the fluorescence signals of opposing ΔF (green and red fluorescence). The ratio signal, collected at both wavelengths, was extracted for the attenuation of any motion artefacts, since motion artefacts can appear as a common change on both wavelengths and consequently they can be cancelled out by the ratiometric calculation^46^. As a result, the introduction of excitation-contraction uncouplers, such as blebbistatin, is not necessary with our system. Blebbistatin may have unwanted effects, such as formation of blebbistatin precipitate, and it may cause changes in metabolic state^47^. Analyses were carried out using a custom-written macro in GraphPad Prism for measurement of action potential duration at 90% of repolarisation (APD_90_) and upstroke duration. Post processing of the raw data included drift removal and normalisation before calculation of AP morphology.

### Synchronisation

During dual modality recordings, the MEA plate was connected to the amplifier of the MEA system and the amplifier was loaded on the stage of the custom made optical mapping system (Fig. 1). The MEA stimulator and the optical mapping camera were connected through a BNC sync cable to generate a blank frame when the stimulator fired. This produced a false stimulation artefact on the optical recording which enabled accurate time correlation between the two recording modalities. MEA recordings were made for 10s and during this time period 5s ratiometric voltage optical mapping recordings were simultaneously obtained.

### Statistical Analysis

Data are expressed as mean ± SEM. Statistical analysis was carried out using GraphPad Prism software (version 6.0 f for Mac OS X, GraphPad Software). Statistical significance was evaluated using student’s t-test (two-tailed), one-way analysis of variance (ANOVA) for unpaired data or ordinary two-way ANOVA followed by Bonferroni multiple-comparison post hoc analysis where appropriate. Baseline and post-modification data are unpaired, unless otherwise stated. The comparison of MEA recordings with optical mapping was validated with linear regression analysis (confidence interval: 95%) for correlation of corresponding characteristics measured with each technique. The APD_90_/FPD, APD_80_/FPD and APD_70_/FPD relationships were compared between the control and modulated data groups by correlation coefficient. All numerical data are presented as mean ± SEM and *p*-value of <0.05 was considered as significant.

### Data availability

All data supporting the findings of this study are available within the article and from the corresponding author on reasonable request.
